# Activation of the Calcium-Sensing Receptor by a Subfraction of Amino Acids Contained in Thyroid Drainage Fluid

**DOI:** 10.1021/acsptsci.3c00350

**Published:** 2024-06-28

**Authors:** Christian Nanoff, Qiong Yang, Roland Hellinger, Michael Hermann

**Affiliations:** † Centre for Physiology and Pharmacology, Gaston H. Glock Laboratories for Exploratory Drug Research, Medizinische Universität Wien, Währinger Straße 13A, Vienna 1090, Austria; ‡ Department of Surgery, Vienna Hospital Association, Klinik Landstraße, Juchgasse 25, Vienna 1030, Austria

**Keywords:** hypoparathyroidism, wound fluid, calcium-sensing receptor, glutamate, acid-sensor, calcilytic drug

## Abstract

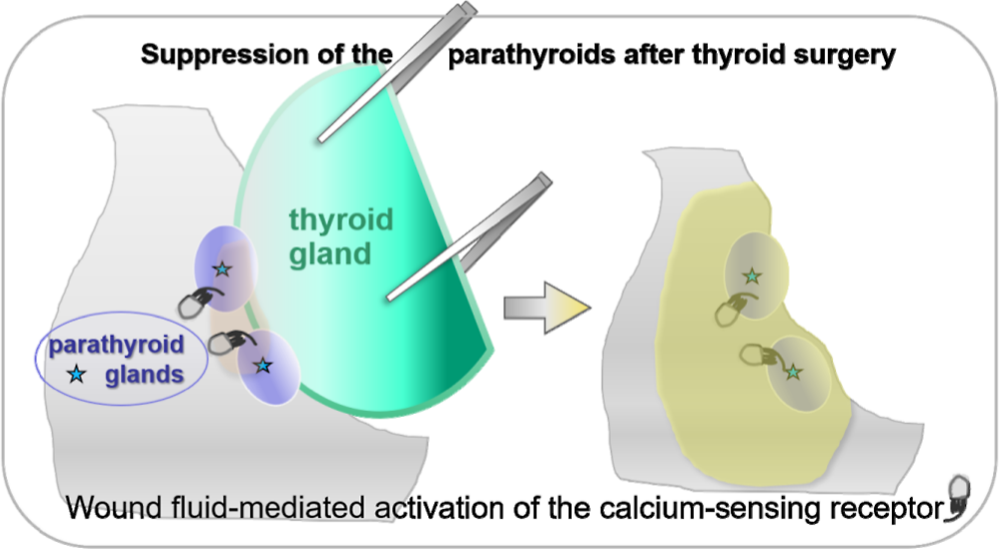

Hypoparathyroidism is a common sequela of thyroid surgery; in this study, we aimed at exploring the pathogenesis behind it. The following premises suggest that wound fluid might be a causative agent. (i) Parathyroid hormone secretion is under feedback control by the calcium-sensing receptor, which responds to a diverse array of activating ligands. (ii) Postoperative hypoparathyroidism arises from a secretory deficiency of the parathyroid glands. Even in patients later unaffected by hypoparathyroidism, parathyroid hormone levels drop within hours after surgery. (iii) Wound fluid is bound to enter the tissue around the thyroid bed, where the parathyroid glands are located. Its composition is shaped by a series of proteolytic reactions triggered by wounding. Using thyroid drainage as a surrogate, we addressed the possibility that wound fluid contains compounds activating the calcium-sensing receptor. Drainage fluid ultrafiltrate was found to be rich in amino acids, and on separation by HPLC, compounds activating the calcium-sensing receptor partitioned with hydrophilic matter that rendered buffer acidic. The data show that glutamate and aspartate at millimolar concentrations supported activation of the calcium-sensing receptor, an effect contingent on low pH. In the presence of glutamate/aspartate, protons activated the calcium-sensing receptor with a pH_50_ of 6.1, and at pH 5, produced maximal activation. This synergistic mode of action was exclusive; glutamine/asparagine did not substitute for the acidic amino acids, nor did Ca^2+^ substitute for protons. NPS-2143, a negative allosteric receptor modulator completely blocked receptor activation by glutamate/aspartate and by fractionated drainage fluid. Thus, wound fluid may be involved in suppressing parathyroid hormone secretion.

Anterior neck surgery puts parathyroid glands at a risk. No other type of surgery is more commonly associated with postoperative hypoparathyroidism, due to the anatomical proximity of the thyroid and the parathyroid glands, than thyroid surgery. After thyroidectomy, up to 25% of the patients develop signs of postoperative hypoparathyroidism, which can be symptomatic hypocalcaemia.^1^ Typically, the level of parathyroid hormone drops early after thyroid resection reaching its lowest concentration within 24 h.^2^ Most patients recover, while in a few, the disorder persists.

Over the years, hypoparathyroidism causing hypocalcemia has remained the most frequent complication of thyroid surgery. Consequentially, preoperative supplementation of calcium and vitamin D has been recommended to prevent hypocalcemia after surgery, though its effectiveness was found to be less than certain.^3−5^ In addition, a surplus of vitamin D can suppress parathyroid hormone secretion further. To come to grips with the disorder (traditionally blamed on physical trauma), research has been directed at identifying risk factors. Variables related to the incidence of acute hypocalcemia were identified in patient’s gender and diagnosis (e.g., Graves’ disease, cancer), and in procedures extending the operation (neck lymph node dissection, parathyroid gland autotransplantation).^1,6^ Among the variables, two were identified as regular hypocalcemia risk factors.^1,6,7^ One is neck dissection required to ensure adequate resection margins around tumor tissue and the draining lymph nodes; this highlights the importance the extent of surgery may have in the development of hypoparathyroidism. The other is a low postoperative level of parathyroid hormone reflecting a secretory deficiency of the parathyroids which is the mechanism underlying postoperative hypoparathyroidism.^2,8,9^ The secretory deficiency predicts a depressed parathyroid hormone level despite low serum calcium, a constellation often found in patients after thyroid surgery.^2,10,11^

The calcium-sensing receptor, a class C G-protein-coupled receptor expressed on the surface of parathyroid cells, mediates feedback regulation. When activated, the receptor lowers parathyroid hormone secretion and thus controls the circulating hormone level. Receptor signal transduction is via the G-protein subtypes G_q_ and G_11_.^12^ Signaling mediated by G_q_/_11_ leads to intracellular mobilization of calcium which represents a corollary of the pathway the calcium-sensing receptor employs to control parathyroid hormone secretion.^13^ The established assay of the receptor typically measures intracellular calcium mobilization triggered by the human receptor orthologue, heterologously expressed in HEK293 cells (HEK-cells).^14^ HEK-cells lack an endogenous complement of the calcium-sensing receptor.^15^

In its recognition of activating ligands, the calcium-sensing receptor is rather unselective. Although the receptor responds to various inorganic cations (Ca^2+^, Mg^2+^, Zn^2+^) and to organic compounds such as spermine, a polyamine and to positively charged (poly)peptides (for example, etelcalcetide, a calcimimetic drug, amyloid-β, and eosinophil cationic protein)^14^ only calcium attains endogenous concentrations (in blood plasma) that mediate receptor activation.^16−18^ In suppressing parathyroid hormone secretion in vitro, the IC_50_ of Ca^2+^ was found to be as low as ∼1.15 mM, commensurate with the physiological range of calcium ions in serum (1.1–1.35 mM).^19^ This tight range is consistent with a steep concentration–response relationship, indicative of a cooperative mode of activation. Its structural basis is in the calcium-sensing receptor homodimer with at least four Ca^2+^-binding sites per monomer.^14^ The receptor sensitivity to calcium ions is subject to allosteric modulation by amino acids and by chloride and phosphate ions.^14,20,21^ Binding of phosphate to the receptor reduces its level of activation, while chloride ions and amino acids enhance the potency of Ca^2+^. A variation in the level of amino acids thus can affect the Ca^2+^-sensitivity of the calcium-sensing receptor.^19,20^ Similarly, extracellular pH modulates receptor sensitivity to calcium ions with small but significant changes in the Ca^2+^-potency,^14^ while a pH of ∼5 by itself was reported to bring about receptor activation.^22^

Wound exudate is known for properties that in principle would render it capable of activating the calcium-sensing receptor. First, wounding lowers tissue pH. Acidification is driven by low oxygen tension in wound fluid and by the metabolic activity of cells involved in the inflammatory reaction and granulation.^23−26^ Second, wound exudate exhibits a proficiency to degrade proteins through the activation of proteolytic enzymes some of which belong to the coagulation and complement cascades.^27,28^ This results in proteolytic activity that cleaves cognate substrates as well as foreign proteins, such as apolipoproteins, serum albumin, and cellular proteins. In this manner, wound exudate draws up its own complement of peptides, which is different from the plasma peptidome on the one hand and distinct when comparing sterile and infected wounds.^29^ Thus, it is a priori possible that the wound exudate generates oligopeptides and releases free amino acids capable of supporting activation of the calcium-sensing receptor.

After thyroid surgery, wound fluid accumulates to measurable volumes.^30^ On postoperative day one, ultrasound-assessed fluid volumes range between 1.5 and 25 mL, while only a minority of the patients have no measurable fluid retention. Wound fluid likely enters the tissue around the resection niche and may expose the calcium-sensing receptor on the parathyroid parenchyma to activating compounds. In this way, wound fluid-dependent receptor activation could suppress parathyroid hormone secretion. If wound fluid underlies the secretory insufficiency of the parathyroids, this would account for (i) the frequency of the incident and (ii) the transient course hypoparathyroidism takes in most of the cases. In addition, (iii) it might establish a link between the extent of surgery and the risk of hypoparathyroidism. Extended thyroid surgery increases exudate volume^31,32^ and a large volume of wound fluid is expected to soak parathyroid parenchyma more effectively than a small volume. Seroma, a tangible accumulation of wound fluid, in fact has been found associated with low levels of serum calcium.^33^ Of note, the placement of a drain fails to significantly reduce the wound fluid volume measured on day one (by sonography) and wound drains in general do not protect from hypoparathyroidism.^27,34^

The aim of this study was to assess the plausibility of the assumption that wound fluid after thyroid surgery is causally related to the risk of postoperative hypoparathyroidism. As a prerequisite to this assumption, the pertinent literature predicts that the postoperative decline of parathyroid hormone levels is no outcome provoked by acute hormonal changes interfering with parathyroid function.^1^ Evaluating patient records from a single surgeon’s uninterrupted case series, we ruled out that other risk factors, but the extent of surgery, impinge on parathyroid hormone levels after thyroidectomy. Then, using thyroid suction drainage fluid, the hypothesis was tested that wound fluid contains activators of the calcium-sensing receptor. Low pH in conjunction with acidic amino acids was found to activate the calcium-sensing receptor, possibly giving rise to a secretory deficiency of the parathyroids.

## Results and Discussion

### Parathyroid Hormone and Serum Calcium Levels after Thyroid Surgery

**Figure 1:**
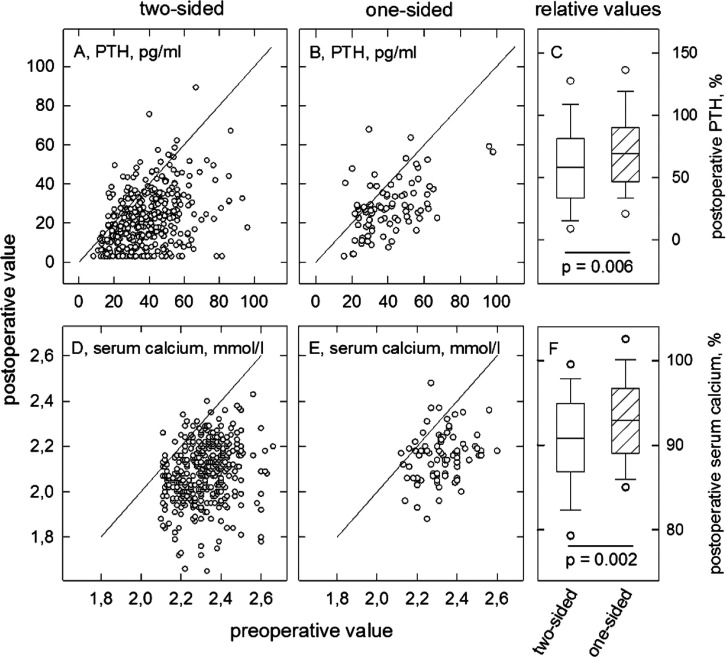
Change of plasma parathyroid hormone (PTH, upper row) and serum calcium levels (bottom row) after thyroid surgery. (A,D) Data plots of patients who underwent two-sided surgery (*n* = 392). (B,E) Data plots of patients subjected to one-sided surgery (*n* = 86). Preoperative values (*x*-variable) are plotted vs postoperative values (*y*-variable), each point representing one patient. In plot A, the lower PTH assay limit (3 pg/mL) curtailed the distribution of data points. (C,F) Percentual change from the respective individual values before surgery. Boundaries of the box indicate the 25th and 75th percentile, respectively, the line within the box, the median value. Error bars mark the 10th and 90th percentile, dots denote the 5 to 95% confidence interval. Open boxes are from patients who underwent two-sided, hatched boxes from those after one-sided surgery. The *p*-values were computed in a Mann–Whitney *U* test.

The records of patients who underwent thyroid surgery document that in most of the instances, the levels of plasma parathyroid hormone and of serum calcium were lower than before surgery. Figure 1 shows plots of parathyroid hormone (Figure 1A,B) and calcium values (Figure 1D,E) where the *x*-axis gives the value before surgery and the *y*-axis the value obtained on postoperative day one, each dot representing an individual patient. Many of the dots scattered below the line of identity (the diagonal). After thyroidectomy (two-sided thyroid surgery), the fraction of patients having a lower value than before surgery was 90% for calcium and 83% for parathyroid hormone (Figure 1A,D). Given interassay coefficients of variation of 2^35^ and 10%^36^ for the measurement of calcium and parathyroid hormone, respectively, values were scored if they were smaller than 0.98 or 0.9 times the individual preoperative values. The analogous data distribution of patients who underwent one-sided surgery is shown in Figure 1B,E. The fraction of patients with values that were lower than those before the operation was 85% for serum calcium and 74% for parathyroid hormone.

### Impact of Extended Surgery

Just as the extent of surgery is related to the incidence of hypoparathyroidism,^7,37^ it was a determinant of the postoperative levels of parathyroid hormone and serum calcium levels. This became apparent from comparing the magnitude of the decrease after two- and one-sided surgery. Figure 1C,F give the serum calcium and plasma parathyroid hormone levels after surgery expressed as percents of the individual preoperative values. They reveal a significantly greater decrease following two-sided than following one-sided surgery. This difference was also seen when concentrations were given as absolute values (Table 1). From the plot shown in Figure 1A, it appears that because of the lower limit of quantification (3 pg/mL), several of the postoperative parathyroid hormone values were an overestimate of their true values. Hence, the decrease in parathyroid hormone levels after thyroidectomy presumably exceeded ∼38% depicted by the box plot (Figure 1C). Clearly, after thyroid surgery (two-sided and one-sided), parathyroid glands are generally prone to some functional deficiency although hypoparathyroidism is common only after thyroidectomy but rare after one-sided surgery.^38^

**Table 1:** Serum Calcium and Plasma Parathyroid Hormone Levels^a^

	sample means (95% confidence intervals)	
	before surgery	day one after surgery
Two-Sided Surgery (*n* = 392)		
serum calcium (mM)	2.31 (2.30–2.32)	2.09 * (2.08–2.10)
plasma parathyroid hormone (pg/mL)	38.9 (37.3–40.5)	22.1 # (20.7–23.5)
One-Sided Surgery (*n* = 86)		
serum calcium (mM)	2.32 (2.30–2.34)	2.16 * (2.14–2.18)
plasma parathyroid hormone (pg/mL)	40.3 (37.0–43.4)	28.2 # (25.3–31.1)

a The difference of postoperative calcium (*) and parathyroid hormone (#) values, respectively, between two- and one-sided surgery was statistically significant (*p* < 0.001, Kruskal–Wallis rank sum test).

The impact the extent of surgery had on postoperative levels was confirmed in a risk model generated by multiple linear regression including variables (patient- and surgery-associated, Table 2) first identified as relevant in an univariable linear regression analysis (Supporting Information Figure S1). The only variable excluded was diagnosis of a carcinoma. Table 2 documents a significant positive correlation between the respective preoperative and postoperative (serum calcium and parathyroid hormone) values and, in addition, depressing effects by parathyroid gland autotransplantation and neck lymph node dissection. Judged by their coefficients, these procedures that extended field and time of the operation had a strong impact on the decrease of the circulating parathyroid hormone. In line with the results reported by Gschwandtner et al., however the number of glands visualized during the operation was of no relevance (Figure S2).^39^ The effect of postoperative parathyroid hormone levels on serum calcium was omitted from the analysis, since after thyroidectomy, serum calcium and parathyroid hormone levels are known to enter into a positive correlation.^2^

**Table 2:** Variables that Predict the Postoperative Level of Serum Calcium and Plasma Parathyroid Hormone, Respectively, Estimated by Multiple Linear Regression Analysis^a^

variable	coefficient	95% confidence interval	*p*-value
Postoperative Serum Calcium			
serum calcium, preoperative	+0.242	0.142, 0.343	<0.001
age	+0.001	0.0002, 0.002	<0.05
female gender	–0.034	–0.064, −0.003	<0.05
parathyroid gland autotransplantation	–0.049	–0.075, −0.022	<0.001
lymph node dissection	–0.063	–0.026, −0.001	<0.001
*R*^2^	0.16		<10^–12^
Postoperative Plasma Parathyroid Hormone			
parathyroid hormone, preoperative	+0.34	0.256, 0.415	<0.001
parathyroid gland autotransplantation	–6.51	–9.34, −3.68	<0.001
lymph node dissection	–6.23	–10.17, −2.29	<0.05
*R*^2^	0.24		<10^–22^

a A likelihood-ratio test based on *F*-statistics was used for assessing the statistical significance of the model (*R*^2^). The significance of the correlation indicated by the coefficient estimate was tested with Student’s *t*-test.

The patient data thus showed that more extensive surgical procedures (one-sided thyroid surgery < two-sided surgery without ancillary surgical procedures < two-sided plus parathyroid autotransplantation/neck dissection) increased the scale of parathyroid hormone and serum calcium decline. Regarding one-sided surgery, it is necessary that the suppressing effect spans an anatomical distance because two intact parathyroid glands are sufficient to sustain calcium homeostasis.^40,41^ Seeping from the site of resection across the midline wound fluid may inhibit a compensatory increase in the level of hormone secretion from the contralateral glands.

### Low Levels of Calcium and Spermine in Thyroid Drainage Ultrafiltrates

In the following section, we tested the hypothesis that material contained in wound fluid supports activation of the calcium-sensing receptor, thus suppressing the parathyroids. An ultrafiltrate of thyroid drainage fluid comprising wound fluid had only low levels of calcium and spermine, the receptor agonists. In an initial sample of 12 patient specimens, calcium concentrations ranged between 0.6 and 1.2 mM (mean = 0.8 mM) and the levels of spermine were between 0.9 and 2.3 μM (mean = 1.2 μM). Either one was below the threshold for activating the calcium-sensing receptor.^14^ As expected for proteolytic reactions contributing to the composition of wound fluid, the ultrafiltrates held concentrations of primary amines including amino acids (mean = 6.2 mM with a range of 1.0–11.4 mM) which in the majority of specimens (9 out of 12) were higher than amino acid levels in blood plasma (∼2.8 mM).^19,20^

**Figure 2:**
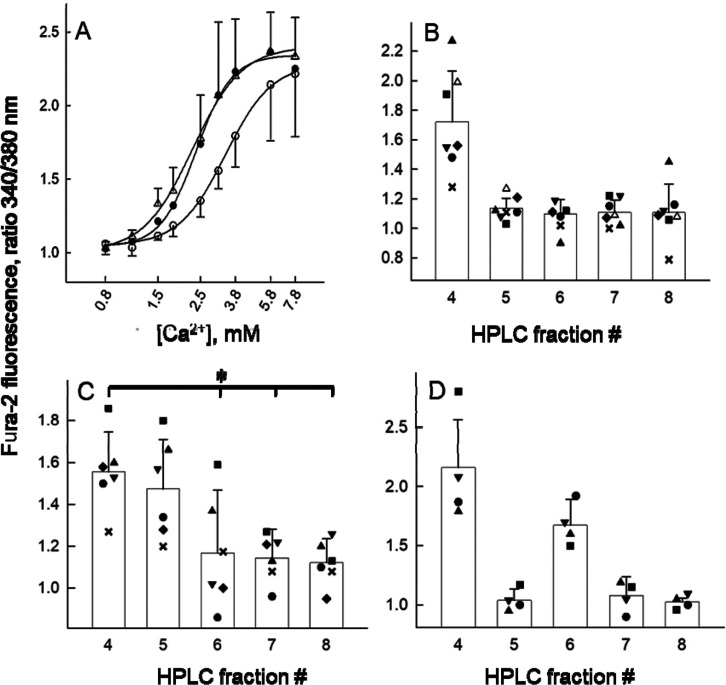
Activation of the calcium-sensing receptor by calcium ions and by fractionated drainage fluid. (A) Ca^2+^-mediated receptor activation (◯, no amino acids added), enhanced by an equimolar mixture of the l-isomers of trp, phe, tyr, his, gln, and ala at concentrations of 2.4 (●) or 4.8 mM (Δ). Given are the ratiometric Fura-2 fluorescence intensity values averaged from five recordings (means ± s.d.). Each calcium concentration series was cumulatively recorded from a single cell-covered glass plate. (B,C) Cellular Fura-2 fluorescence intensities recorded with HPLC-fractions four through eight. Shown are individual fluorescence values of fractionated specimens represented by graphic symbols (one symbol per specimen) and their means (±s.d., bars with whiskers). (B) *n* = seven thyroid drainage fluid specimens. Fraction four intensity values were significantly different from each of the values recorded with fractions five through eight (*p* < 0.05 computed by Dunn’s method that followed a Kruskal–Wallis ANOVA on ranks). (C) *n* = six breast drainage specimens. A post hoc Holm–Sidak test indicated that the difference in fluorescence values was significant between fraction four vs fractions six, seven and eight (*, *p* < 0.05). (D) Cellular fluorescence intensities recorded with fractionated casein hydrolysate (*n* = 4 HPLC-separations of casein hydrolysate), represented as in (B,C).

The experiment shown in Figure 2A served to gauge the synergistic effect caused by the combination of amino acids with threshold Ca^2+^ in the receptor activation assay. In the presence of an equimolar mixture of tryptophan, phenylalanine, tyrosine, histidine, alanine, and glutamine (all l-isomers) at concentrations of 2.4 and 4.8 mM the calcium concentration–response curves (EC_50_ ∼ 2.3 mM) separated from the curve recorded with no amino acids added (EC_50_ ∼ 3.4 mM) and the calcium threshold dropped to lower values. Amino acids at 4.8 mM and also at 2.4 mM enhanced the fluorescence recorded with 1.5 mM Ca^2+^ which in the absence of amino acids was indistinguishable from the baseline (Figure 2A). The inclusion of the aromatic amino acids delineated the maximum possible enhancement of the receptor response when calcium is at its threshold level.^20^ Thus, it was unlikely that the combination of calcium ions with amino acids contained in the ultrafiltrate is sufficient to cause activation of the calcium-sensing receptor. In fact, immersion of receptor expressing cells in the ultrafiltrate gave only spurious effects in three instances (Figure S3A).

### Activation of the Recombinant Calcium-Sensing Receptor by a Subfraction of Organic Matter in Drainage Fluid Ultrafiltrate

Conceivably suction drainage fluid represents an adulterated version of the wound fluid. Hematoma was a regular contaminant, and the collected volumes were between 15 and 80 mL, resulting in variable dilutions (by serum and/or plasma) of wound fluid. However, a concentrating step (by HPLC on a reversed-phase C18 column) allowed for the enrichment of organic compounds activating the calcium-sensing receptor. The calcium ions present in the drainage samples were largely washed out with the column void volume. The eluate that followed was separated in five consecutive fractions (labeled four to eight). On lyophilization of the eluate, the dried matter was taken up in a small volume of assay buffer to give a solution about 5-fold concentrated over the original ultrafiltrate. Figure S3B depicts Fura-2 excitation wavelength scans recorded from cells exposed to fraction four sample fluid from three individual specimens. The Fura-2 340/380 nm ratio was 1.90 for the sample. An assay carried out with the addition of NPS-2143 (1 μM), the negative allosteric modulator of the calcium-sensing receptor, gave a 340/380 nm ratio of 1.17. With a baseline ratiometric Fura-2 fluorescence of 1.0 (±0.1) which was recorded from the same cells exposed to the assay buffer alone, a major portion of the effect was receptor-specific.

Figure 2B summarizes the assay results (340/380 ratios) after the HPLC-fractionation of ultrafiltrates from seven individual drainage specimens. As depicted by the bar diagram the material endowed with intrinsic activity exclusively eluted in fraction four, i.e., at isocratic conditions, with the mostly aqueous mobile phase. The 340/380 nm ratios varied between specimens in a range of 1.28 to 2.23. Their averaged ratio was significantly greater than those recorded with each of the later fractions.

Given their weak retention on the reverse-phase C18 matrix, the compounds eluting in fraction four were hydrophilic. When applied as external standards, spermine came out in fraction four, whereas tryptophan eluted in fraction seven. Though enriched above its level in drainage ultrafiltrates, the concentrations of spermine in fraction four were between 3 and 9 μM and below 30 μM, the estimated receptor activation threshold (Figure S4).

### Receptor Agonists Present in Wound Fluid and Casein Hydrolysate

If the receptor-activating principle is a product of protein degradation, it needs not be exclusive to thyroid drainage fluid. Intrinsic activity was also present in drainage fluid collected after breast surgery (Figure S5A) and, in addition, in casein hydrolysate. As with thyroid drainage fluid, an unequivocal demonstration of receptor activation required fractionation and enrichment by HPLC (for breast drainage fluid, see Figure S5B). The averaged Fura-2 fluorescence intensity values (340/380 nm ratios, Figure 2C,D) revealed that compounds with intrinsic activity from breast drainage fluid and from casein hydrolysate similarly distributed to fraction four (and with some but lesser propensity to another fraction). Given its weak retention on the reverse-phase C18 matrix, the material contained in fraction four was considered rather hydrophilic.

### Fraction Four Intrinsic Activity Not Associated with Oligopeptides

The comparison with the casein hydrolysate was indicative of amino acids and possibly of oligopeptides comprising the active principle. Oligopeptides, however, were unlikely candidates for the following reasons. First, an assessment of fraction four samples by MALDI-TOF/TOF-MS failed to identify peptides with a molecular mass >700 Da. MALDI-MS detected few signals attributable to very short peptide species with low signal intensities in fractions four from drainage fluid and casein hydrolysate alike (Figure S6). Second, oligopeptides known to activate the calcium-sensing receptor typically carry many positive charges.^14^ While a ten-meric designer peptide (RRRRKRVNTK) activated the calcium-sensing receptor at concentrations as low as 25 μM, pentameric RINKK (*M*_r_ = 658 Da), the only strictly polybasic sequence encoded by any one of the bovine casein genes was inactive even when tested at concentrations up to 10 mM (Figure S7). Since oligopeptides were ruled out, the assumption was that thyroid drainage fluid and casein hydrolysate comprised a homologous subfraction of amino acids endowed with intrinsic activity.

### Comparison of Fractions Four from Thyroid Drainage Fluid and Casein Hydrolysate: Time-Dependent and Concentration-Dependent Activation of the Calcium-Sensing Receptor

**Figure 3:**
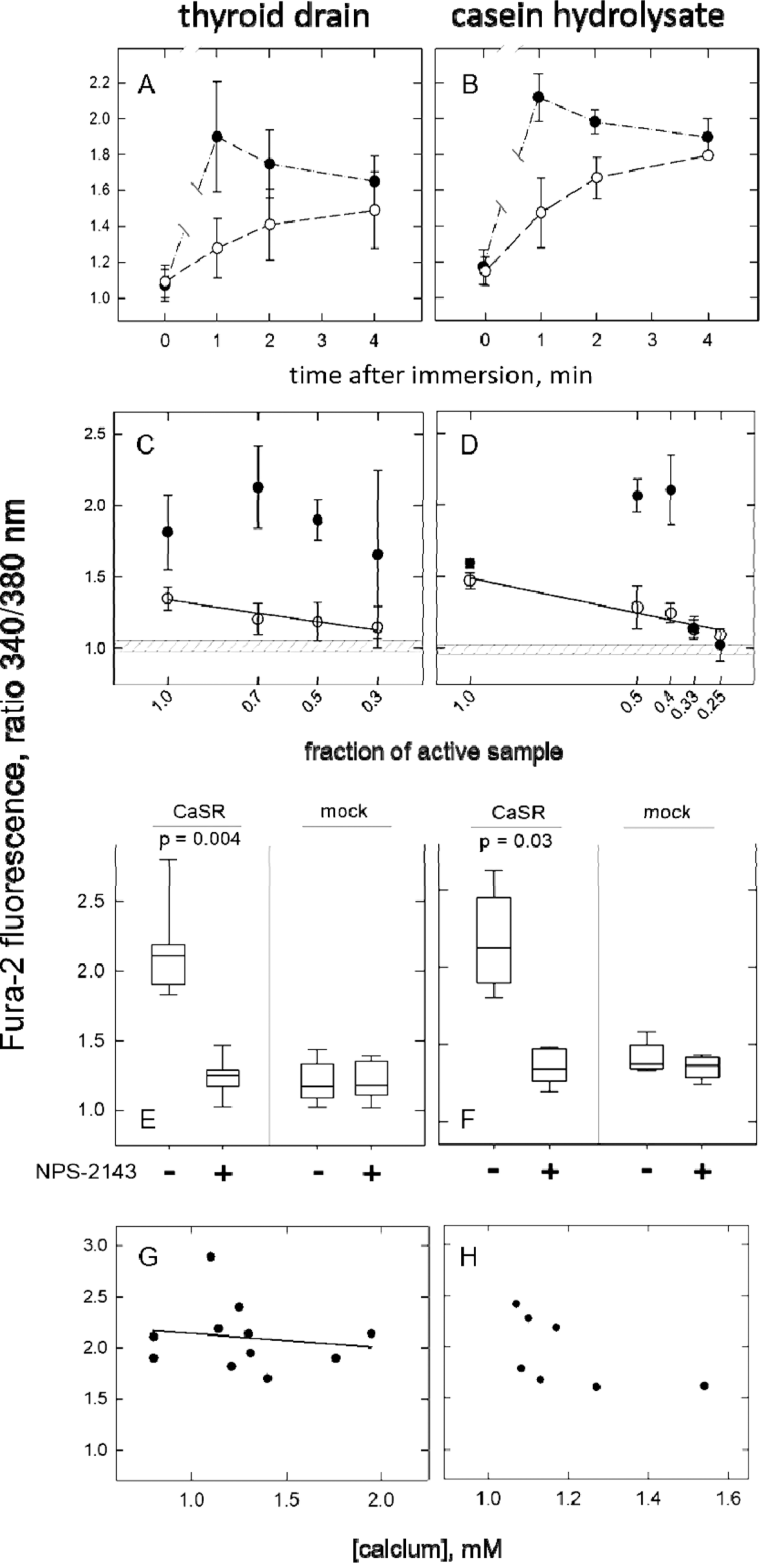
Ca^2+^-independent activation of the calcium-sensing receptor by a fraction of hydrophilic amino acids from thyroid drainage specimens (left column of figures) and from casein hydrolysate (right column of figures). (A,B) Time course of Fura-2 fluorescence intensity changes recorded from receptor-expressing cells (●) or from nontransfected HEK-cells (◯) exposed to fraction four material. Ratiometric Fura-2 fluorescence values averaged from recordings (±s.d.) carried out with 11 drainage fluid specimens (A) and 4 recordings with fraction material from casein hydrolysate (B). A break in the connecting lines indicates that prior to 1 min fluorescence intensities were found higher than at 1 min. (C,D) Sequential dilution of fraction four. One minute Fura-2 recordings from receptor-expressing cells (●) and nontransfected cells (◯), respectively, exposed to the indicated dilutions (made in assay buffer) of fraction four material. Each recording was from a separate cell-covered glass plate. Decimal numbers (*x*-axis) indicate the dilution factors. Data points represent means (±s.d.) from 5 to 11 drainage fluid specimens each (C) and three fractions of casein hydrolysate (D), respectively. The hatched field delineates the 95% confidence interval of the Fura-2 fluorescence from the same cells exposed to assay buffer, which was similar in transfected and nontransfected cells. Straight lines represent linear regression of the data from nontransfected cells (◯). (E,F) Inhibition by NPS-2143 of the effect elicited by exposure to fraction four material. One minute fluorescence values were recorded at the optimal sample dilution from receptor-expressing and nontransfected HEK-cells, respectively, with or without the addition of NPS-2143 to the sample (1 μM), each from a separate cell-covered glass plate (E, drainage specimens *n* = 11; (F) casein hydrolysate *n* = 4; error bars represent 95% CI). The *p*-values were computed by a Tukey-test comparing data from all four categories that followed a Kruskal–Wallis ANOVA on ranks. (G) Concentration of calcium ions present in the sample subjected to the Fura-2 assay (documented in E). Plot of the calcium concentration vs the 1 min Fura-2 fluorescence value, data points grouped along the regression line. (H) Shows an analogous plot encompassing individual active fractions from casein hydrolysate assayed at various dilutions.

Despite the different origins and presumably different compositions of the materials, their effects were remarkably similar. For the comparison, fraction four samples with appreciable activity (*n* = 11) were selected out of an expanded set of thyroid drainage specimens (collected from a later series of patients). In Figure 3, the left-and the right-hand panels represent the data obtained with thyroid drainage and casein hydrolysate fraction four materials, respectively. The time course of the effect (Figure 3A,B) demonstrates receptor-specificity at early time points when receptor-stimulated Fura-2 fluorescence intensity (●) was most pronounced. The decay of fluorescence intensity that ensued was a typical pattern also observed if calcium or spermine was used to activate the calcium-sensing receptor (Figure S8). In nontransfected HEK-cells (◯) fluorescence intensity was low at 1 min but rose steadily such that after 4 min the difference between receptor-expressing cells and HEK-cells devoid of the receptor became minimal. The reversibility of this receptor-independent effect decreased successively as indicated by the Fura-2 fluorescence intensity measured after immersing the cells in wash buffer (=assay buffer) for 5 min. If cells had been incubated in sample fluid for 4 min or longer, after the subsequent wash-out phase Fura-2 fluorescence intensity was 1.14-fold (±0.09, mean ± s.d.) higher than the value at baseline whereas after a 2 min incubation in sample fluid followed by wash-out, fluorescence intensity returned to baseline values.

The effectiveness of fraction four suggests an enrichment of the active principle, and conversely, dilution reduced the ability of fraction four to activate the receptor. Figure 3C,D (●) show that sequential dilution with assay buffer caused a sharp decline of activity. From individual samples in Figure 3C, intrinsic activity was lost following a small dilution step, similar to that shown in Figure 3D (dilution of casein hydrolysate samples). However, the averaging of the data obtained with individual patient samples (Figure 3C) seems to extend the concentration-dependence. Notably, Figure 3C,D suggest that the first dilution step increased the fluorescence intensity relative to the undiluted sample. In contrast, recording from nontransfected HEK-cells cellular Fura-2 fluorescence intensity decreased proportionally to sample dilution (◯, connected by a regression line).

Fraction four material from drainage fluid ultrafiltrates when dissolved in assay buffer had a pH-value between 4 and 7, from casein hydrolysate the pH was <4. Thyroid drainage material eluting into fractions five and higher did not lower the pH to <7.2. Based on evidence presented in Figure 5, the dilution-dependent decrease in receptor activation was attributable, in part, to increasing pH. Yet, very acidic conditions (plotted at *x* = 1.0 in Figure 3C,D) impaired receptor activation relative to the conditions obtained after dilution in buffer (plotted at *x* = 0.7 in Figure 3C, at *x* = 0.5 in Figure 3D).

### Inhibition of the Effect of Fraction Four Sample Fluid by NPS-2143

Figure 3E,F summarize the effect of NPS-2143, the negative allosteric modulator of the calcium-sensing receptor (1 μM). The box plots document the averaged Fura-2 fluorescence intensities obtained with fraction four from thyroid drainage material (Figure 3E) diluted to maximize receptor activation (Figure 3C). In receptor-expressing cells, the mean of the 340/380 nm ratios was 2.1. NPS-2143 reduced the fluorescence signal to 1.2, a value similar to the signal produced by fraction four in nontransfected cells. Figure 3F shows the results obtained with fractions prepared from casein hydrolysate, which were similar to those with the thyroid drainage specimens.

### Fraction Four Residual Calcium

Although most of the calcium ions eluted with the flow-through (60 mL, corresponding to virtual fractions one to three) from the HPLC column, some calcium was also retrieved in fraction four. Figure 3G gives the calcium levels measured in fraction four from the individual thyroid drainage samples plotted against the respective one min Fura-2 signals (y-variable). Figure 3G,H show that the calcium concentrations mostly were below the receptor-activating threshold; in Figure 3G only the highest level at ∼2 mmol/L (1.2 mmol/L plus 0.8 mmol/L calcium from the assay buffer) would according to the results shown in Figure 2A produce some receptor activation if added alone. For the thyroid drainage data set as a whole, the regression line indicates no correlation between the contaminating calcium and the receptor-stimulated Fura-2 fluorescence. Thus, the sample characteristics illustrated in Figure 3A,C,G point to acidic amino acids (rather than to calcium ions) conferring receptor activation.

### Summary of Receptor Activation by Patient Drainage Samples

**Figure 4:**
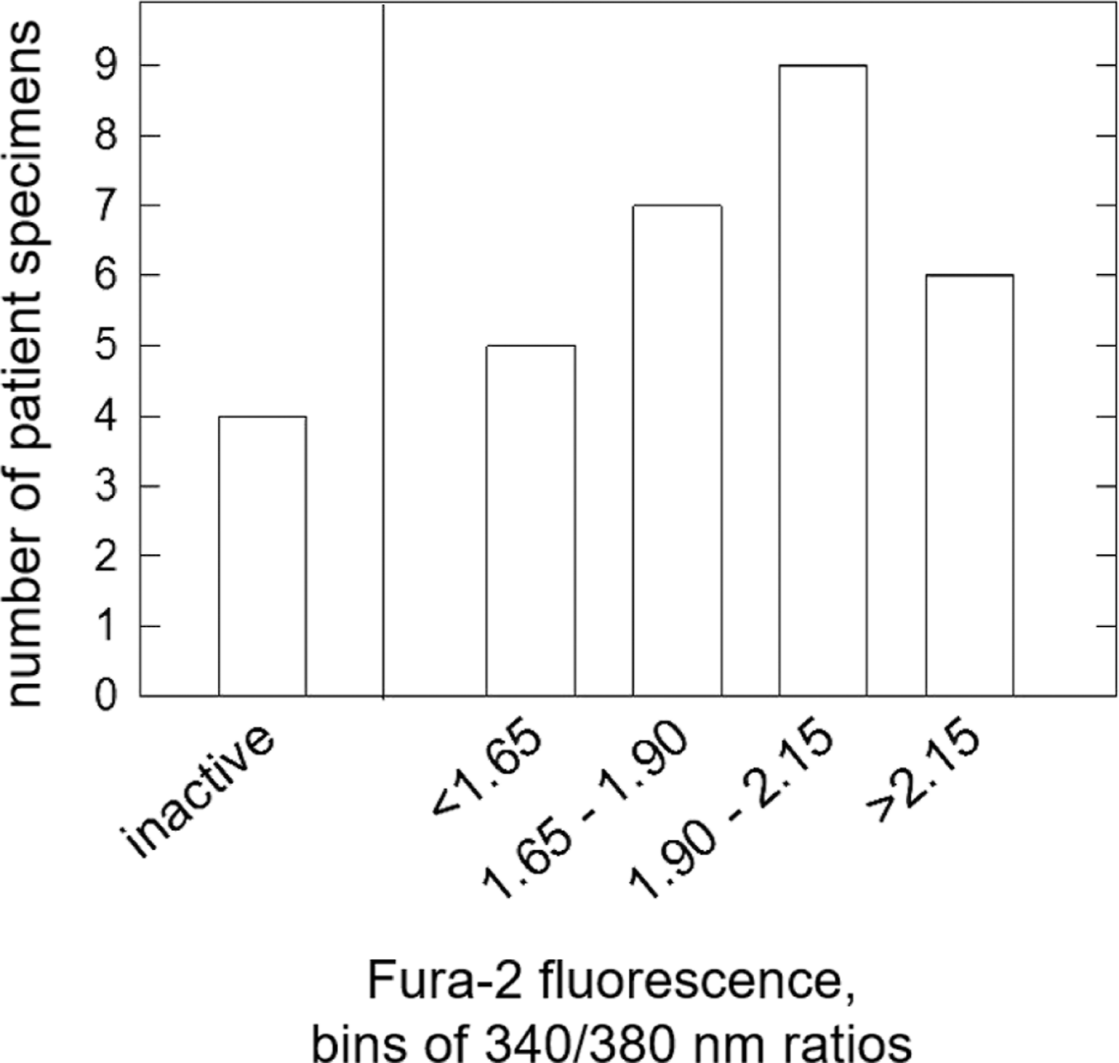
Activation of the calcium-sensing receptor by individual thyroid drainage fluids (*n* = 31) after fractionation. Distribution of 1 min Fura-2 fluorescence values according to binned intensities. Samples were scored inactive if the response of receptor-expressing cells was indistinguishable from the response recorded from nontransfected HEK-cells.

A total of 31 drainage specimens provided evidence that the ability of fractionated drainage fluid to activate the calcium-sensing receptor was a common feature. Figure 4 shows the number of patient specimens where fraction four caused higher Fura-2 fluorescence intensity in receptor-expressing cells than in nontransfected HEK-cells; the individual data are given in Table S1. Only 4 of the patient samples were found to be inactive. In spite of the small sample size, the percentage of samples demonstrated to activate the calcium-sensing receptor was comparable to the percentage of patients with postoperative levels of calcium and parathyroid hormone lower than their preoperative values.

### Activation of the Calcium-Sensing Receptor by Glutamate and Aspartate

**Figure 5:**
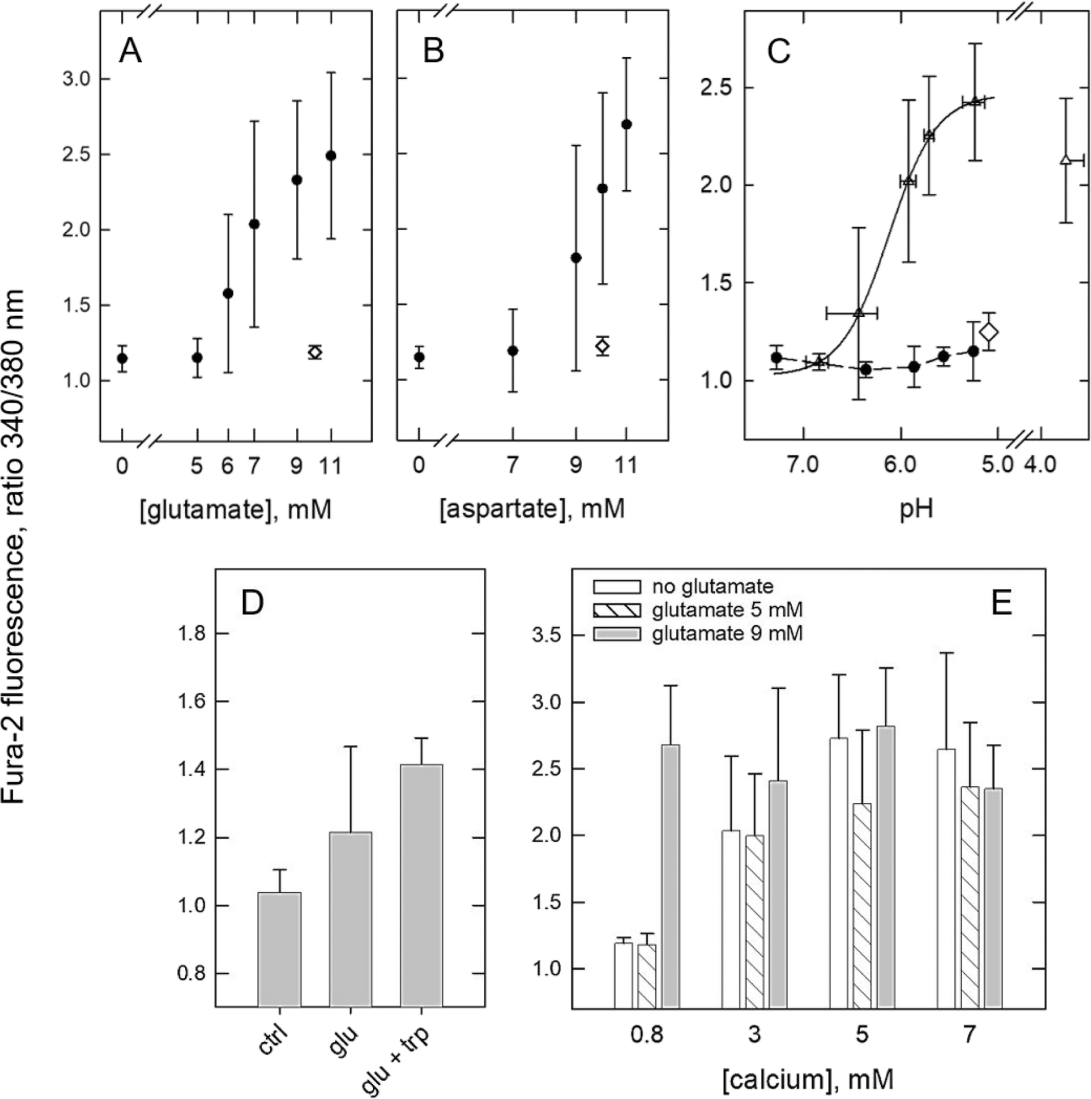
Activation by protons of the calcium-sensing receptor exposed to glutamate or aspartate (recordings done 1 min after start of the exposure). (A,B) Concentration-dependent receptor activation by l-glutamate (A) and l-aspartate (B). At the plotted concentrations the sample pH values were in (A) 6.8 at 5 mM; 6.6 at 6 mM; 6.4 at 7 mM; 5.8 at 9 mM; 5.0 at 11 mM glutamate, and in (B) 6.7 at 7 mM; 6.1 at 9 mM; 5.2 at 10 mM; 4.7 at 11 mM aspartate. Diagram (A) depicts Fura-2 fluorescence intensity recorded with 10 mM glutamine (◊), diagram (B) Fura-2 fluorescence intensity recorded with 10 mM asparagine (◊). (C) pH-dependent change of Fura-2 fluorescence intensity recorded with (Δ) or without (●) the addition of glutamate (9 mM); pH was adjusted with isotonic buffers containing Hepes or Mes (plus NaCl and 0.8 mM CaCl_2_) and measured for each sample. The horizontal error bars indicate binning of samples with similar pH values; bins represent the standard deviations of the means. Each value in figures A to C is the means of 3 to 8 determinations (±s.d.). The effect of 10 mM glutamine at pH 5.1 is represented by an open diamond symbol (◊, *n* = 3, mean ± s.d.). The spline curve was obtained by fitting the data between pH 5.2 and 6.8 to a Hill-equation. (D) Neither acidic pH nor tryptophan was capable of enhancing receptor activation by glutamate at its threshold concentration (5 mM). Cellular Fura-2 fluorescence recorded after immersion of cells in buffer (ctrl, *n* = 8), after the addition of glutamate (5 mM, *n* = 5) or of glutamate (5 mM) plus tryptophan (3 mM, *n* = 3). Final assay pH always was 5.6. (E) Effect of the addition of glutamate at 5 mM (hatched bars) or at 9 mM (gray bars) on Ca^2+^-mediated activation of the calcium-sensing receptor. Each bar represents the mean (±s.d.) of 3 to 5 recordings. Recordings ± glutamate were obtained in parallel (open bars represent recordings in the absence of glutamate) with each recording from a separate cell-covered glass plate.

Similar to amino acids, gamma-glutamyl tripeptides are designated positive allosteric modulators of the calcium-sensing receptor.^14^ Between published reports the potency estimates for glutathione differ over a wide range, from 80 nM to millimolar concentrations.^42−44^ In our assay, glutathione activated the calcium-sensing receptor at concentrations greater than 3 mM (Figure S9). This effect was emulated by glutamate, a constituent amino acid of glutathione, and similarly by aspartate. Figure 5A,B shows the effects of glutamate and aspartate, respectively, in the receptor-dependent calcium mobilization assay. The plots of the evoked Fura-2 fluorescence signals revealed an abrupt concentration-dependent onset of the effect with maximal activation occurring at concentrations ≥10 mM. A comparison with Figure 2A suggests that glutamate and aspartate caused full receptor activation. By contrast, glutamine and asparagine, the amidated biosynthetic precursor of glutamate and metabolite of aspartate, respectively, at 10 mM (◊) had no effect. Activation of the calcium-sensing receptor by the acidic amino acids relied on the proton concentration. Glutamate, aspartate, and similarly glutathione when dissolved in the Hepes assay buffer lowered the pH. At glutamate and aspartate concentrations from 5 to 11 mM the pH was between 6.8 and 5.0.

### Synergistic Effect of Acidic Amino Acids with Protons in Activating the Calcium-Sensing Receptor

Figure 5C demonstrates that conversely, a low proton concentration decreased the odds of receptor activation by glutamate (9 mM). For the pH-dependent assay of receptor activation, Hepes was replaced with Mes buffer adjusted to acidic pH. Since the lower end of the pH series borders the Mes buffering range, the pH value of each individual sample was measured with a pH electrode. In Figure 5C, the individual sample pH-value is plotted against cellular Fura-2 fluorescence intensity (Δ). Horizontal error bars represent bins of samples with similar pH. Fitting the fluorescence data to a Hill equation returned a pH value of 6.1 necessary for half-maximal activation (pH_50_); the Hill-coefficient was 1.8. At a pH of ∼5 receptor activation was near-maximal. Further acidification to pH < 4 caused a decline in Fura-2 fluorescence intensity, which mirrors the increment in Fura-2 fluorescence produced by the first dilution step applied to fraction four samples (Figure 3C,D). In contrast, buffer acidification alone (Figure 5C, ●) was not sufficient to produce a change in the cellular Fura-2 fluorescence. At pH 5.1, the addition glutamine to a final concentration of 10 mM caused an insignificant increment in Fura-2 fluorescence (◊).

Thus, the results shown in Figure 5A–C indicate a synergistic effect exerted by glutamate/aspartate together with protons in activating the calcium-sensing receptor, a conclusion corroborated by the following findings. First, the concentration–response relationships for glutamate (Figure 5A) and aspartate (Figure 5B) were steeper than those obtained with protons in the presence of a fixed concentration of glutamate (Figure 5C). The likely explanation is that in the experiments shown in Figure 5A,B, the sample pH decreased concomitantly with increasing concentrations of glutamate/aspartate. This intensified synergistic activation the higher the amino acid concentration. Since the p*K*_a_- and p*K*_b_-values of glutamate and aspartate fall outside the covered pH range, probably the effect is through the active receptor conformation stabilized by protons.

Second, as exemplified by the inability of glutamine to replace glutamate in receptor activation (Figure 5C), the combination partners responsible for a synergistic mode of activation were not interchangeable. (i) Tryptophan acts as a positive allosteric modulator in Ca^2+^-mediated receptor activation but failed to substitute for the acidic amino acids. At pH 5.6, the combination of 5 mM glutamate with tryptophan (3 mM) barely increased Fura-2 fluorescence intensity relative to glutamate alone (Figure 5D). (ii) Figure 5E shows that glutamate at its threshold (5 mM) was unable to enhance receptor activation by calcium ions. Vice versa, calcium ions failed to elevate 5 mM glutamate above the receptor-activating threshold: the bars in Figure 5E representing the Fura-2 fluorescence intensities recorded without (open bars) or with 5 mM glutamate in the assay buffer were similar at each calcium concentration. Conversely, there was no enhanced glutamate-dependent receptor activation (at 9 mM) when assayed in the presence of increasing concentrations of calcium ions (Figure 5E, gray bars).

### Activation by Protons of the Calcium-Sensing Receptor at 37 °C

The interaction with glutamate as well as the ability to recognize the level of protons are reported properties of the calcium-sensing receptor.^14,19,20^ A previous publication had demonstrated the ability of the calcium-sensing receptor to act as pH sensor with no auxiliary action by calcium ions or amino acids.^21^ As shown in the paper and recapitulated here—using prewarmed assay buffer and a water-thermostat cuvette holder—at 37 °C, the receptor responded to protons when their concentration exceeded 3 μM (pH < 5.5). However, at assay temperatures between 22 and 27 °C (room temperature), the same concentration of protons failed to activate the receptor if glutamate was absent (cf. Figure 3C).

An assay temperature raised to 37°, however, had only a moderate effect on receptor activation by Ca^2+^. It weakly increased sensitivity to calcium ions, but not to glutamate. The EC_50_ for Ca^2+^ was 2.6 mM at 37° compared to 3.3 mM at room temperature (Figure S10A). The glutamate threshold concentration was similar to that at room temperature; at 7 mM, glutamate gave a significant increase in the Fura-2 fluorescence intensity (Figure S10B). Thus, the elevated assay temperature enhanced receptor sensitivity to proton-mediated activation.

### Specificity for the Calcium-Sensing Receptor of the Effect Elicited by Protons Together with Acidic Amino Acids

**Figure 6:**
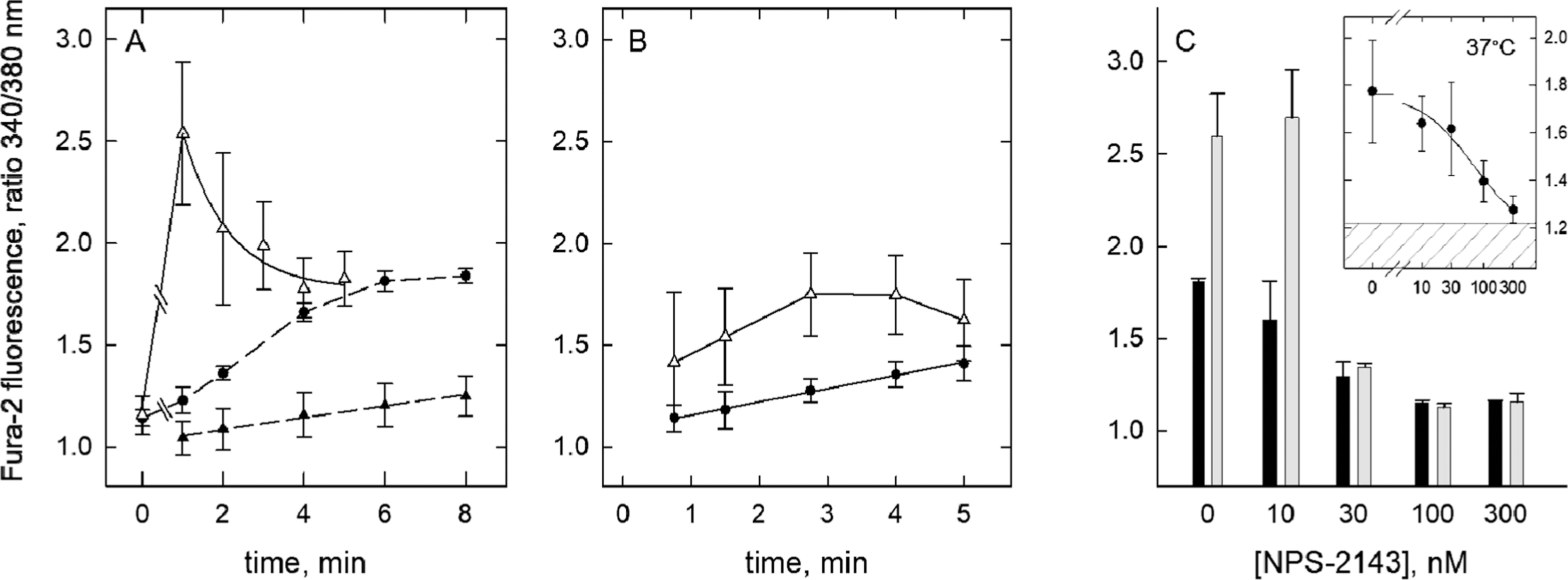
Receptor-specificity of the effect on Fura-2 fluorescence by protons with or without glutamate. (A) Recording at room temperature. Time-resolved change of Fura-2 fluorescence recorded from nontransfected HEK-cells (●) and from cells expressing the calcium-sensing receptor (Δ), either challenged with 9 mM glutamate at pH 5.8. Time 0 represents averaged recordings with no glutamate added, at neutral pH. In addition, Fura-2 fluorescence was recorded from receptor-expressing cells exposed to acidic buffer alone at pH 5.6 (▲, slope of the regression line = 0.008 ratio units/min). Data are means (±s.d.) from five recordings each. The spline curve depicting the decline of the glutamate-triggered Fura-2 fluorescence represents the graph of an exponential decay computed by data fitting (decay constant = 0.26/min). (B) Recording at 37 °C. Time-resolved change of Fura-2 fluorescence recorded from receptor-expressing cells immersed in buffer at pH 5.1. Shown are averaged recordings (*n* = 3 to 5) obtained in the absence (Δ) or the presence of NPS-2143 (0.3 μM, ●, slope of the regression line = 0.065 ratio units/min), following a 5 min preincubation with NPS-2143 at room temperature. The initial lag phase observed in the absence of NPS-2143 (Δ) varied between assays, likely to do with warming of the cell substrate. (C) Bar graph. Fura-2 fluorescence intensity recorded (at room temperature) from receptor-expressing cells 1 min after the addition of glutamate (11 mM, gray bars) or of calcium (4.8 mM, black bars). Cells were preincubated with NPS-2143 at the indicated concentrations for 5 min before the addition of glutamate or calcium. The assay buffer pH was adjusted such that its value was ∼5.1 after the addition of glutamate and calcium, respectively. The final concentration of DMSO, the NPS-2143 solvent, was 0.1% in all instances. Results are the means (±s.d.) of three recordings each. The Figure inset shows concentration-dependent inhibition by NPS-2143 of receptor activation by protons (pH = 5.1). Recordings were done at 37 °C and 2.75 min after immersion of cells in prewarmed buffer at pH 5.1. Given are the means of three recordings (±s.d.). The hatched area represents the 95% confidence interval of the baseline values recorded (in assay buffer at pH 7.4 and 37°) from the same cells used for assessing the inhibition by NPS-2143.

Assays carried out on nontransfected HEK-cells and assays with the use of NPS-2143 confirmed that the proton-driven effect at room temperature and at 37°, respectively, was mediated by the calcium sensing receptor. Figure 6A presents data recorded at room temperature showing the time-resolved effects of glutamate (9 mM, pH = 5.8) on Fura-2 fluorescence in cells expressing the calcium-sensing receptor (Δ) and in nontransfected HEK-cells. They were similar to those produced by fraction four samples (cf. Figure 3A,B) further indicating that acidic amino acids are the receptor coactivators present in fraction four. Challenging the calcium-sensing receptor with low pH plus glutamate (Δ) caused an early peak in fluorescence intensity which leveled out thereafter. Exposure of nontransfected HEK-cells to glutamate, however, gave a slow rise in Fura-2 fluorescence intensity attaining a maximum level within 6 min after the addition of glutamate (●). The effects depicted here for glutamate were repeated with aspartate at 10 mM (Figure S11). A control assay using acidic buffer (pH = 5.6) alone revealed that at room temperature even longer exposure to protons alone had only a minor effect on cells expressing the receptor (Figure 6A ▲), which was similar to nontransfected HEK-cells (Figure S12).

The mechanism of glutamate/aspartate increasing Fura-2 fluorescence intensity in nontransfected HEK-cells is undefined. It is probably initiated by proton-driven uptake of sodium glutamate/aspartate for which ASCT2 is a transmembrane carrier.^45^ This may account for the protracted effect that tended to become persistent: at late time points (6 min and later), only about 75% (±7%) of the glutamate-increment in Fura-2 fluorescence disappeared after a 5 min wash-out (*n* = 4). When wash-out was initiated after one min, its effect was complete (97 ± 4%, *n* = 3). The increase in Fura-2 fluorescence intensity is presumably driven by an ion exchange mechanism introducing calcium to the cell interior. However, ASCT2 is not expressed based on RNA and protein data in parathyroid cells^46^ where this receptor-independent effect therefore is unlikely.

Figure 6B shows Fura-2 fluorescence values recorded at 37 °C without the addition of acidic amino acids. If the proton concentration was raised to ∼8 μM (pH 5.1) the receptor-expressing cells responded with an increase in Fura-2 fluorescence intensity, as reported.^22^ Time course and peak effect size clearly differed from those observed at room temperature (when challenged with glutamate or aspartate at acidic pH, Figures 6A and S11) but as expected, the effect was blocked by concomitant exposure to NPS-2143 (0.3 μM, ●).

At neutral pH, NPS-2143 inhibited Ca^2+^-mediated receptor activation with an IC_50_ of ∼0.5 μM (Figure S13). While this value is in the range of reported IC_50_ estimates,^14^ it does not accurately predict the block provided by 0.3 μM NPS-2143 at acidic pH (Figure 6B). Figure 6C shows inhibition by NPS-2143 of the glutamate effect (at 11 mM, gray bars) in comparison with inhibition of the Ca^2+^ effect (at 4.3 mM, black bars), both assessed at pH ∼ 5.1 and room temperature. Prior to recording, the cells were preincubated with NPS-2143 for 5 min to allow for its association with the receptor. The bar graph represents the averaged fluorescence intensity values, recorded in the presence of NPS-2143 at the indicated concentration. It becomes obvious that inhibition by NPS-2143 was maximal at 100 nM and that 100 nM NPS-2143 fully suppressed the effects of Ca^2+^ and of glutamate, respectively. A difference between Ca^2+^ and glutamate showed in the sloping decrease of fluorescence intensity which was gradual on receptor activation by Ca^2+^ but steep in the presence of glutamate. Despite this discrepancy, the sensitivity to inhibition by NPS-2143 at low pH appeared exquisite in either mode of activation. An IC_50_ estimate of ∼20 nM (with Ca^2+^ as the agonist) suggests that acidity increased the inhibitory potency of NPS-2143.

A similar finding was made at 37° and pH 5.1. The fluorescence intensities recorded 2.75 min after immersion of cells in pH 5.1 buffer at 37° are shown in Figure 6C inset. The spline curve represents a fit of the data to an equation describing hyperbolic decay from a maximum with an IC_50_ estimate of ∼60 nM. As it is unlikely that at pH 5.1 the NPS-2143 molecule accepts a proton, the acidity must have an impact on the NPS-2143 binding site of the calcium-sensing receptor.

### Testable Hypothesis to Account for the Secretory Deficiency of the Parathyroid Glands

It appears reasonable to presume that the early (i.e., within the first 24 h) drop of parathyroid hormone levels occurs as a result of the same mechanism that underlies postoperative hypoparathyroidism; the risk for either one is determined by the extent of thyroid surgery. The high incidence rates together with the transient course the disorder takes in most of the instances plus the observation that parathyroid hormone levels decrease even after one-sided surgery all converge on the assumption that thyroid surgery creates a common and reversible effect suppressing the parathyroids. In the present study, we aimed at exploring wound fluid-mediated activation of the calcium-sensing receptor, the receptor that relays feedback inhibition and controls parathyroid hormone secretion.

Wound fluid generates tissue acidosis and is replete with protein degradation products. Proteolysis has been reported to result from the activity of an array of enzymes, which eventually leads to the release of amino acids. Measurements in microdialysates in fact confirmed a surge of free amino acids in acute experimental wounds^47,48^ and in keeping, their levels were elevated in drainage fluids relative to those detected in blood plasma or serum. A local excess of amino acids would support the activation of the calcium-sensing receptor by calcium ions; however, the thyroid niche lacks a source from which extra calcium could spill into the wound fluid.

Conversely, the experimental data presented here are consistent with an alternative mechanism of receptor activation that arises from tissue acidosis. It has long been known that wounds shortly after infliction produce an acidic milieu triggered by low oxygen tension, by the consumption of buffering bicarbonate and the accumulation of lactate.^23,49^ Low pH has been found repeatedly in acute wounds^50−52^ and, therefore, it appears counterintuitive that the pH of drainage fluid was at or above neutral. The reason for this is unclear but may be attributable in part to the presence of hematoma as blood and serum have marked buffering capacity. Hence, a limitation to our line of arguments is that the acidity and molar composition of wound fluid entering the parathyroid glands may be difficult to determine in ex vivo assays. As inferred from the activation properties of acid sensing ion channels, tissue acidosis is expected to span a pH range between 7 and 5. A proton level of 10 μM approaches—but does not exceed—maximal activation of acid-sensing ion channels in peripheral nerves and thus overlaps with the pH-dependent activation range of the calcium-sensing receptor.^53^ Despite some caveat, the present evidence does not rule out but rather supports a wound fluid-based mechanism underlying parathyroid secretory deficiency. In the absence of a suitable animal model, a critical test of the involvement of the calcium-sensing receptor would require the trial of a calcilytic drug in patients undergoing thyroid surgery. Since low pH fails to impair receptor inhibition by NPS-2143, the prototype of calcilytic drugs, such a trial should be feasible and would examine an opportunity for the prevention of postoperative hypoparathyroidism.

## Methods

### Postoperative Change in Plasma Parathyroid Hormone and Serum Calcium Values

We analyzed postoperative changes recorded in two groups of consecutive patients who underwent thyroid surgery, each operation performed by the same surgeon. Group one included 392 patients who underwent bilateral lobectomy (two-sided surgery) between 2007 and 2012 (81% female, age range = 14–89 yrs., mean = 56 yrs.). Group two comprised 86 patients subjected to a one-sided operation (thyroid lobectomy or enucleation) during the same period (71% female, age range 17–88 yrs., mean = 55 yrs.). A previous study by Gschwandtner et al.^39^ reported a part of the group one patient data. The frequency distribution of diagnoses (goiter, thyroiditis, Graves’ disease, and cancer) in their study and ours therefore is comparable. No one of the patients was operated for hyperparathyroidism. The diagnoses of patients from group two were goiter (95% of the cases), thyroiditis, and reoperation for Graves’ disease, respectively. Data collection and the procedure defining two-sided surgery followed the details as outlined in^38^ who also specified the methods employed for the determination of calcium and parathyroid hormone (specific for the intact peptide), respectively, from patient plasma/serum.

In these operations, parathyroid autotransplantation was carried out according to an ad-hoc decision that followed prespecified criteria.^54^ The one-sided operations were performed as outlined by Promberger et al.^38^ Differing from the studies published by Gschwandtner et al. and Promberger et al.,^38,39^ the only inclusion criteria applied was that patients were from an uninterrupted case series and had records documenting plasma parathyroid hormone and serum calcium values measured before surgery and on day one after surgery. Patient care followed routine surgical procedures; therefore, no other of the hormones impinging on calcium homeostasis (25-hydroxyvitamin D, calcitonin, fibroblast growth factor 23, the soluble form of Klotho, PTH-related peptide) was determined from patient blood plasma/serum. Records did not require documentation of the postoperative course pertaining to patient recovery, the development of hypocalcemia, or other outcomes. Prior to surgery, patients received no prescription for calcium or vitamin D supplements. For the purpose of the present study, which was to assess the individual changes in hormone and calcium levels, we also considered records with preoperative parathyroid hormone values above the standard range (17 of the patients before bilateral surgery and two before unilateral surgery had values between 67 and 96 pg/mL). The preoperative levels of serum calcium were within the normal range of 2.1–2.7 mmol/L.

According to the guidelines issued by the Vienna Hospital Association, this type of study required no approval from the institutional ethics board. Patients gave written and informed consent to the scientific evaluation of the per-protocol data.

### Statistical Analysis

We confirmed the normal distribution of the serum calcium and plasma parathyroid hormone values across the patient sample using chi-square test statistics. A difference between subgroup means (according to the presence or absence of a binary variable) was assessed by the Mann–Whitney rank sum test. Between-gender differences in the distribution of variables were evaluated with chi-square statistics. To analyze the impact of potential risk factors, we employed a linear regression model with postoperative values (plasma parathyroid hormone or serum calcium) as outcome variables. Fitting the data to a multiple linear regression returned estimates of the regression coefficients (β_1_ + β_2_, ...., + β_*i*_ where β_*i*_ is the regression coefficient attributed to the variable *x*_*i*_). Individual *x*-variables were continuous (age, preoperative values of parathyroid hormone or calcium, parathyroid gland autotransplantation) or binary (gender, diagnosis, lymph node excision). Reported are only those that correlated to the outcome variables in a statistically significant manner. The regression coefficient estimates were tested for statistical significance by *t*-test and the probability that the linear model was appropriate by a log likelihood ratio test (F-statistics). The tests used to estimate the statistical significance of differences between assay results are given in the figure and table legends.

### Processing of Suction Drainage Fluid

Closed suction drainage is routinely employed after thyroid surgery at the Department. The University ethics committee approved the use of drainage fluid for experimental purpose. Drainage flasks were retrieved within 24 h after surgery and were immediately processed in the laboratory. The drainage fluid usually contained hematoma. After centrifugation and sedimentation of the particulate matter, we cleared the sample supernatant by ultrafiltration passing the fluid through a polycarbonate membrane with a nominal cutoff value of 10.000 Da (Merck Millipore, Germany). The resulting filtrate had no hue and was virtually devoid of large-size proteins, and its tonicity was in a range between 150 and 350 (on average 251) mosm/L. The procedure was the same for suction drainage specimens collected from patients who underwent breast surgery at the Department of Surgery, Medical University of Vienna.

For HPLC, a Kromasil reverse-phase C18 column (dimensions 250 × 21.2 *ø* mm, 10 μm) was used operated on a Dionex Ultimate 3000 system. Chemicals were obtained from Carl Roth, Germany. The mobile phase consisted of 5% acetonitrile with 0.1% (v/v) trifluoroacetic acid in water (eluent A) and 90% (v/v) acetonitrile with 0.1% (v/v) trifluoroacetic acid in water (eluent B). We applied ultrafiltrate (8 to 12 mL with a solid matter average of 7 mg/mL) acidified with 0.1% trifluoroacetic acid as counterion. Individual drainage fluid specimens were subjected separately to chromatography. Separation started with a washing step with 60 mL of eluent A after application of the sample and was completed with an acetonitrile-gradient in a volume of 120 mL to a maximum of 90% acetonitrile (eluent B). The collection of the eluate continued for a volume of 100 mL, whereafter the UV-absorbance (wavelength 214 nm) trace returned to baseline. The collected fractions (20 mL each) were dried by lyophilization. In the same manner, we fractionated casein hydrolysate (Fluka Biochemika/Merck Life Sciences) applying ∼100 mg of dry matter dissolved in the mobile phase solvent. Until experimental use, all samples were kept at −20 °C and for the assay were taken up in buffer (assay buffer consisting of Hepes NaOH 20 mM, pH 7.4, NaCl 125 mM and CaCl_2_ 0.8 mM) resulting in a clear solution. For buffer at acidic pH values, Mes (2-(*N*-morpholino)ethanesulfonic acid) replaced Hepes as necessary. All chemical reagents were of analytical grade and were obtained from Merck Life Sciences (Germany).

### Composition of Drainage Fluid Ultrafiltrates

The calcium concentration of drainage fluid ultrafiltrate was determined by photometry using cresolphtalein as complexation reagent according to a formulation proposed by Moorehead and Biggs.^55^ For the quantification of primary amines, we used a ninhydrin reagent. In brief, 0.25 mL of sample fluid added to 1 mL of ninhydrin solution (2.5% w/v ninhydrin in 95% DMSO with 0.5% v/v acetic acid) was heated to 70° for 10 min. After dilution with 2 mL of 70% v/v ethanol, absorbance was measured at 570 nm, and the values were converted to millimolar concentrations using alanine solutions as the standard. Spermine (and spermidine) was quantified from lyophilized thyroid drainage fluid ultrafiltrate and from fractionated material by liquid chromatography–tandem mass spectrometry exactly as described by Magnes et al.^17^ The protein content of the ultrafiltrates was determined with a Bradford reagent using bovine serum albumin (solution supplied by the manufacturer) as a reaction standard. For the measurement of the osmotic concentration, we used a vapor pressure osmometer; the standard solutions contained 100, 290, and 1000 mM NaCl.

MALDI-TOF/TOF-MS analysis of the peptides was performed with an Autoflex MALDI-TOF MS analyzer (Bruker Daltonics). All spectra were acquired in positive reflector mode. The mass spectrometer was calibrated routinely with five-point calibration using a quadratic function in the internal calibration mode with a Peptide Mix 4 from LaserBiolabs (Sophia-Antipolis, France). The matrix, α-cyano-hydroxy-cinnamic acid (10 mg/mL), dissolved in acetonitrile/ddH_2_O/TFA 50/50/0.1% (v/v/v), was mixed with the sample at a ratio of 6:1. For the analysis, the dried droplet method was used with 0.5 μL aliquots of the mixture spotted on an MTP384 ground steel target plate. Summed spectra were acquired, processed, and compared to reference spectra derived from only matrix to identify analyte signals. Mass spectrometry equipment along with reagents required for operation is described in ref (56).

### Activation of the Recombinant Calcium-Sensing Receptor

The cDNA encoding the human calcium-sensing receptor was from Deutsches Ressourcenzentrum für Genomforschung and made available by Dr. E. Helen Kemp (University of Sheffield, UK). The sequence encodes a naturally occurring mutation replacing arginine with glycine at position 990 of the receptor polypeptide. For the transfection of mammalian cells, the receptor-coding sequence was inserted into the pcDNA3.1 expression plasmid, where gene expression is under the control of a viral promoter. We verified the correct insertion by fluorescent sequencing. As a host cell line, we used HEK293 cells (HEK-cells). We confirmed that HEK-cells lack endogenously expressed calcium-sensing receptors as Ca^2+^ had no effect on Fura-2 fluorescence in nontransfected cells. Cells stably expressing the recombinant receptor were raised in culture. We limited the time of uninterrupted cultivation to 2 weeks and replenished the live culture from frozen stocks of mass-cultivated cells positive for the selection marker (resistance to G418, Geneticin).

For the assay of receptor activation, cells grown on poly-d-lysine-covered glass plates were loaded with the calcium-binding fluorophore Fura-2 during a 2 h incubation at room temperature in assay buffer with 2 μM Fura-2-AM. Signal transduction by the activated calcium-sensing receptor leads to intracellular calcium mobilization which causes increased Fura-2 fluorescence. Assays of receptor activation by drainage fluid samples were done by submersing cell-covered glass plates into the clear sample fluid and recording Fura-2 fluorescence in a Hitachi F4500 spectrofluorometer essentially as described by.^22^ Recording of Fura-2 fluorescence was routinely carried out at room temperature in assay buffer (consisting of Hepes.NaOH 20 mM, pH 7.4, NaCl 125 mM, and CaCl_2_ 0.8 mM). The instrument scan captured the fluorescence signal from an ensemble of cells determined by the width of the light beam. The ratio of Fura-2 fluorescence emission (at 510 nm) recorded on excitation at wavelengths of 340 and 380 nm was used for quantitation. This ratiometric assay of calcium-dependent fluorescence intensity has the advantage of sufficient reproducibility that within limits is independent of the amount of fluorophore taken up by the cells. Baseline fluorescence was recorded from cells submersed in assay buffer, where the calcium concentration (0.8 mmol/L) was below the receptor-activation threshold. We routinely confirmed the response of the receptor-expressing cells to an agonist by measuring the effect of Ca^2+^ (4.1 mM). Receptor-specificity of the signal was assessed with nontransfected HEK-cells and with the use of a specific receptor inhibitor, NPS-2143 (2-chloro-6-[(2*R*)-2-hydroxy-3-[(2-methyl-1-naphthalen-2-ylpropan-2-yl)amino]propoxy]benzonitrile, Selleck Chemicals, Germany), respectively. Fura-2 fluorescence recorded from receptor-expressing cells in assay buffer, i.e., the baseline fluorescence signal, was completely insensitive to NPS-2143.

## Materials

Peptides generously provided by Dr. Horst Ahorn (Boehringer-Ingelheim Austria, Vienna, Austria) were synthesized by the solid-phase method. Additional oligopeptides were obtained from piChem (Grambach, Austria). Fura-2-AM, amino acids, spermine, cell culture reagents, salts, and buffer reagents were obtained from Merck.

## Supporting Information Available

Serum calcium (left) and plasma parathyroid hormone (PTH) levels on day one after thyroidectomy; stratification of the patient sample according to the number of parathyroid glands visualized in situ; excitation wavelength scans; activation of the calcium-sensing receptor by spermine; and excitation wavelength scans (PDF)
