# Supplementary material for: Activation of the Calcium-Sensing Receptor by a Subfraction of Amino Acids Contained in Thyroid Drainage Fluid
Source: ACS Pharmacol Transl Sci. 2024 Jun 28;7(7):1937–50. doi: 10.1021/acsptsci.3c00350 (PMC11249632; doi:10.1021/acsptsci.3c00350)
Supplement: Supplementary file 1 — pt3c00350_si_001.pdf [file pt3c00350_si_001.pdf]

Supporting Information

Activation of the calcium-sensing receptor by a subfraction of amino acids contained in thyroid drainage fluid

Christian Nanoff[1]*, Qiong Yang[1], Roland Hellinger[1] and Michael Hermann[2]

[1] Medizinische Universität Wien, Centre for Physiology and Pharmacology, Gaston H. Glock Laboratories for Exploratory Drug Research, Währinger Straße 13A, 1090 Vienna, Austria

[2] Vienna Hospital Association, Klinik Landstraße, Department of Surgery, Juchgasse 25, 1030 Vienna, Austria

* Corresponding author (christian.nanoff@meduniwien.ac.at)

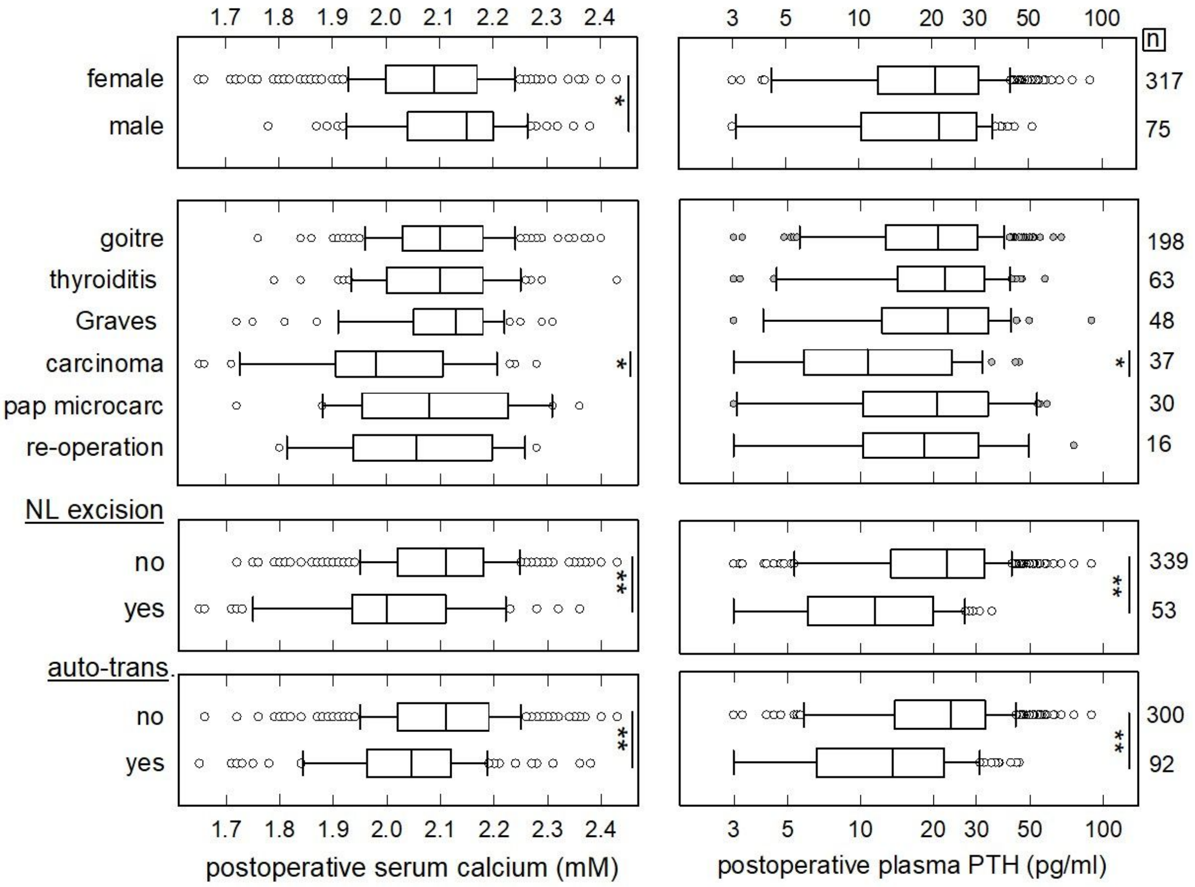

Figure S1. Serum calcium (left) and plasma parathyroid hormone (PTH) levels (right) on day one after thyroidectomy. Stratification of the study population by gender, diagnosis and ancillary surgical procedures (NL = lymph node, auto-trans. = parathyroid gland auto-transplantation). Values are represented as box plots where the left and right boundaries of the box indicate the 25th and 75th percentile, respectively, the line within the box the median value. Error bars represent the 10th and 90th percentile, dots individual outliers. Parathyroid hormone values are plotted on a logarithmic scale to better visualize the low range of values. Asterisks indicate groups (female, carcinoma, lymph node excision, parathyroid gland auto-transplantation) significantly correlated with postoperative calcium or parathyroid hormone on univariable regression analysis. ★ indicates $p < 0.05$, ★★ $p < 0.001$. Although the parathyroid hormone concentration after surgery was indistinguishable between men and women, there was a significant difference in serum calcium levels. This might arise from a sex-specific component in parathyroid hormone action, for which experimental evidence exists[57].

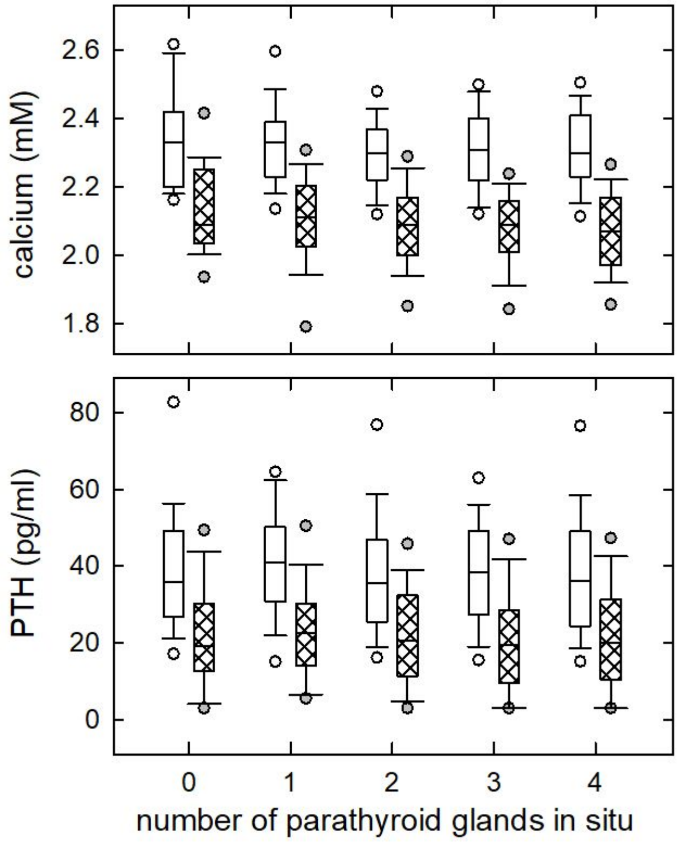

Figure S2. Stratification of the patient sample according to the number of parathyroid glands visualized in situ. Serum calcium (top) and parathyroid hormone values (PTH, bottom) before (open box) and after (hatched box) thyroidectomy (two-sided thyroid surgery). Boundaries of the box indicate the 25th and 75th percentile, respectively, the line within the box the median value. Error bars mark the 10th and 90th percentile, dots the 5th and 95th percentile. In each number-of-glands-category, the differences between pre- and postoperative values were significant (ANOVA followed by Dunn's test for multiple comparisons). The values recorded before and after surgery, respectively, were not different between categories. The number of patients with zero glands visualized was 21; with 1 gland 62; with 2 glands 142; with 3 glands 120; with four glands 47.

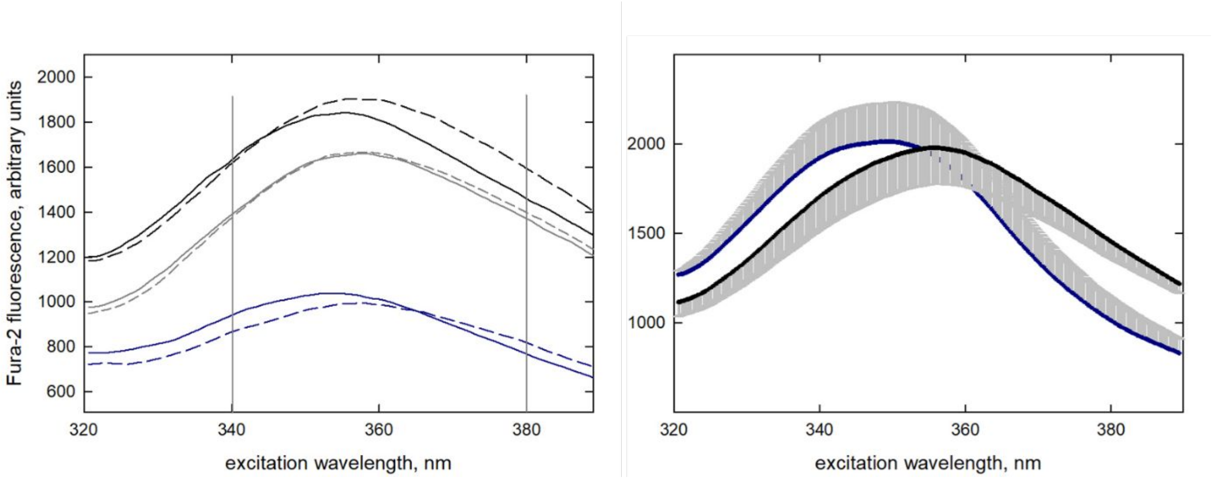

Figure S3 A) Excitation wavelength scans recorded from cells exposed to thyroid drainage fluid ultrafiltrate before (solid line) and after the addition of NPS-2143 (1 μM, dashed line), each from a separate cell-covered glass plate. Trace lines represent the fluorescence intensity measured at 510 nm. Recordings were acquired one minute after the immersion of cells in sample fluid. Three individual specimens (black, grey and blue) are shown. The vertical lines intersect the fluorescence trace at excitation wavelengths of 340 and 380 nm, respectively. B) Excitation wavelength scans recorded from cells exposed to HPLC-fractions four of thyroid drainage fluid ultrafiltrates. Shown are averaged trace lines acquired with fraction four samples from three specimens (blue line, ratio 340/380 nm = 1.90). The black line represents the mean values of the same samples after the addition of NPS-2143 (1μM), ratio 340/380 nm = 1.17. For the sake of clarity, only positive or negative error bars representing s.e.m. values are depicted.

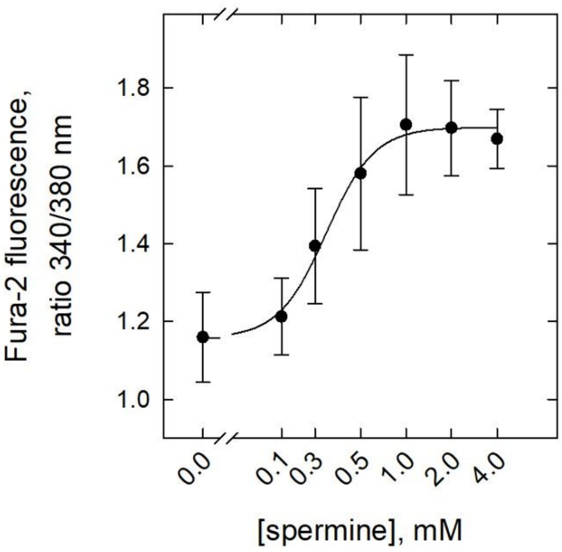

Figure S4. Activation of the calcium-sensing receptor by spermine. Fura-2 fluorescence intensity was cumulatively recorded from receptor-expressing cells in the presence of increasing concentrations of spermine in assay buffer ([Ca^{2+}] = 0.8 mM). Fluorescence recordings were acquired two minutes after the addition of spermine from a 1000-fold concentrated stock solution. Shown are means (± s.d.) of 5 recordings. Curve fitting returned an EC_{50} value of 0.3 mM and a Hill-coefficient of 2.1.

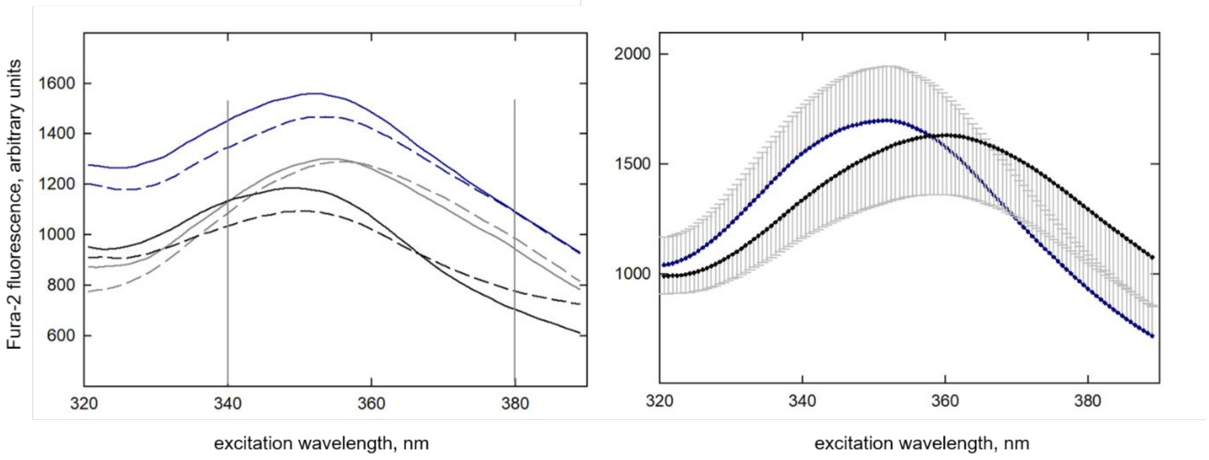

Figure S5 A) Excitation wavelength scans recorded from cells exposed to breast drainage fluid ultrafiltrate before (solid line) and after the addition of NPS-2143 (1 µM, dashed line), each from a separate cell-covered glass plate. Trace lines represent the fluorescence intensity measured at 510 nm. Recordings were acquired one minute after the immersion of cells in sample fluid. Three individual specimens (black, grey and blue) are shown. The vertical lines intersect the fluorescence trace at excitation wavelengths of 340 and 380 nm, respectively. B) Excitation wavelength scans recorded from cells exposed to HPLC-fractions four of breast drainage fluid ultrafiltrates. Shown are averaged trace lines acquired with fraction four samples from three specimens (blue line, ratio 340/380 nm = 1.67). The black line represents the mean values of the same samples after the addition of NPS-2143 (1µM), ratio 340/380 nm = 1.05. For the sake of clarity, only positive or negative error bars representing s.e.m. values are depicted.

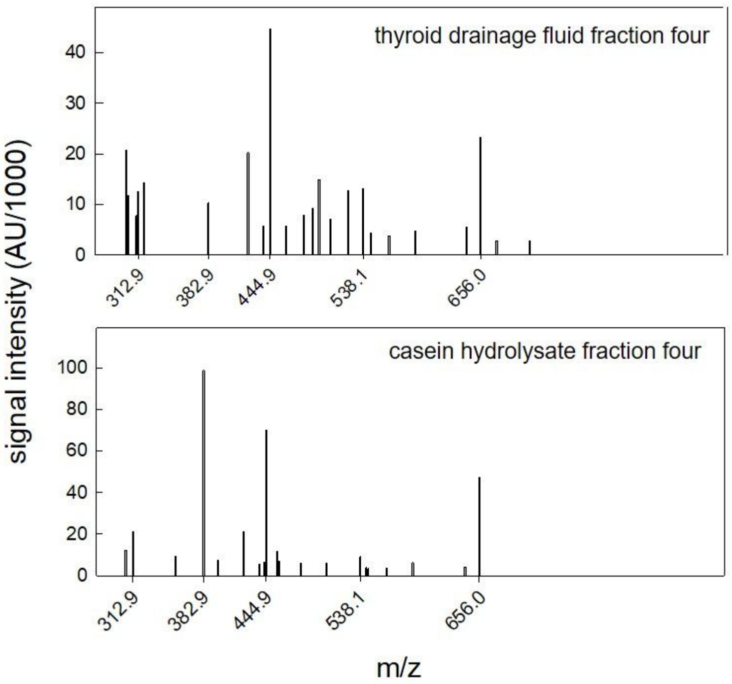

Figure S6. Representative MALDI-MS signals recorded in fraction four samples, obtained by HPLC separation of thyroid drainage ultrafiltrate (top panel) or casein hydrolysate (bottom). Shown is a graphic rendering of the signal m/z and intensity values. The signal traces were blank-corrected by eliminating signals recorded from the matrix without sample (from replicate recordings). Labels on the x-axis indicate m/z values of signals detected in both thyroid drainage and casein hydrolysate samples. Subsequent MS/MS analysis of drainage samples identified only a single tetrameric peptide with a tentative amino acid sequence of DVxx, which indicates that the origin of all other signals was non-peptidergic compounds. No significant additional signals were detected in fraction four samples from two other specimens.

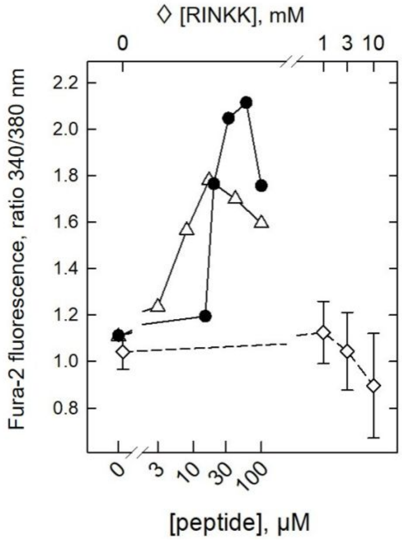

Figure S7. Activation of the calcium-sensing receptor by polybasic peptides. Fura-2 fluorescence intensity values recorded one minute after the addition of an 18-mer designer peptide (single letter code sequence: RRRRKRVNTKRSSRAFRAH) (Δ) or of a ten-mer designer peptide (RRRRKRVNTK) (●), at concentrations indicated at the bottom x-axis. Shown are mean values of duplicate determinations, each recording from a separate cell-covered glass plate. The top x-axis (including a line break) gives the concentrations of RINKK (◊), a pentameric peptide (sequence encoded by the bovine beta-casein gene). The symbols represent means (± s.d.) of three one-minute recordings.

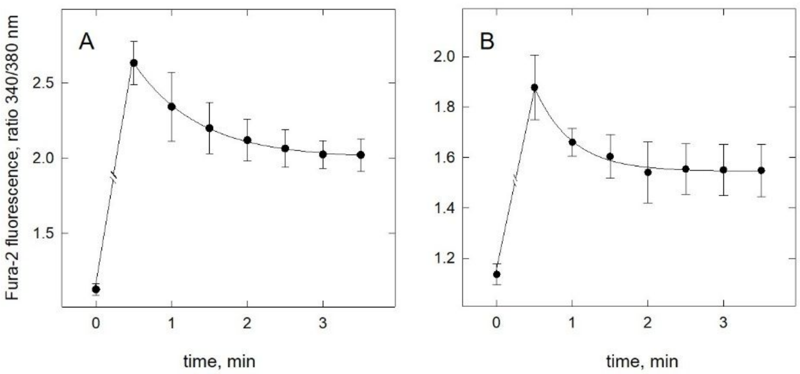

Figure S8. Time course of Fura-2 fluorescence intensity following activation of the calcium-sensing receptor by Ca^{2+} (3.8 mM, A) or spermine (0.5 mM, B). Calcium chloride or spermine was added to the cuvette with a cell-covered glass plate immersed in assay buffer under gentle stirring. Recordings were done before (time 0) and at the indicated time points after the addition of calcium chloride or spermine in

volumes that were 1% the assay volume. The spline curve connecting the data point at minute 1 with the later time points was obtained by fitting the data to a function describing exponential decay. Fitting returned decay constants of 1.1/min (Ca^{2+}) and 2.0/min (spermine). Data are means (± s.d.) from three recordings.

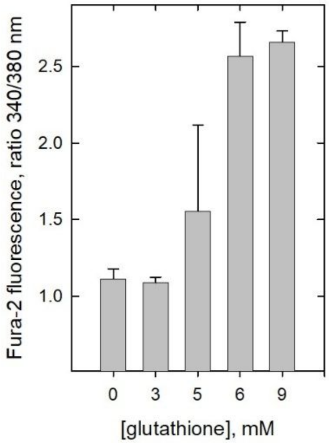

Figure S9. Activation by glutathione of the calcium-sensing receptor (Fura-2 fluorescence recordings made one minute after start of the exposure). The sample pH values were 7.2 at 3 mM, 6.8 at 5 mM, 6.6 at 6 mM, 5.2 at 9 mM glutathione. Shown are means (± s.d.) of at least three recordings, each from a separate glass plate.

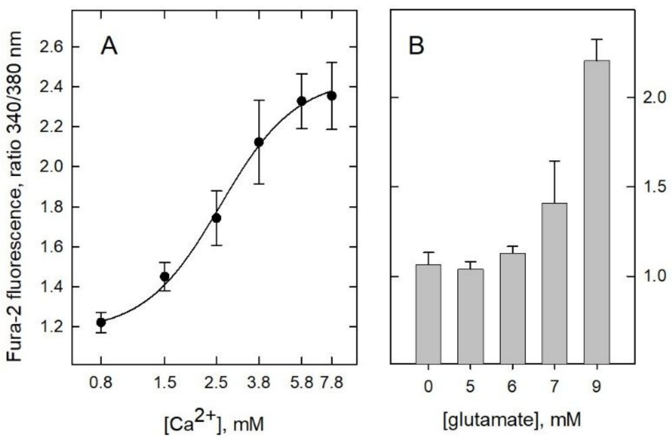

Figure S10. Cellular Fura-2 fluorescence recordings carried out at 37°C. A) Ca^{2+}-mediated receptor activation. Given are the ratiometric Fura-2 fluorescence intensity values averaged from five recordings (means ± s.d.) with cumulative addition of Ca^{2+} to the indicated concentrations. Fitting the averaged data to a Hill-equation returned an EC_{50} estimate of 2.7 mM. B) Glutamate-mediated activation of the calcium-sensing receptor. At the plotted concentrations the sample pH values were 6.8 at 5 mM; 6.6 at 6 mM; 6.4 at 7 mM; 5.8 at 9 mM glutamate. Bars represent the averaged Fura-2 fluorescence intensity values (± s.d.) from three recordings, each performed on a separate cell-covered glass plate, one minute after the addition of glutamate. A Dunn's post-hoc test confirmed that the fluorescence intensity recorded with 7 mM was significantly higher ($p = 0.01$) than the controls, recorded in the absence of glutamate.

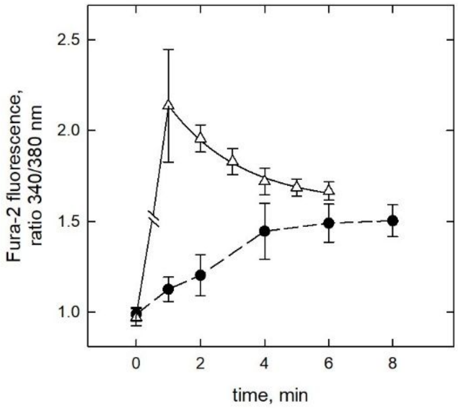

Figure S11. Time-resolved change of Fura-2 fluorescence recorded from non-transfected HEK-cells (●) and from cells expressing the calcium-sensing receptor (Δ),challenged with 11 mM aspartate at pH 4.7. Time 0 represents averaged recordings with no aspartate added, at neutral pH. The spline curve depicting the decline of the aspartate-triggered Fura-2 fluorescence represents the graph of an exponential decay computed by data fitting (decay constant = 0.44/min).

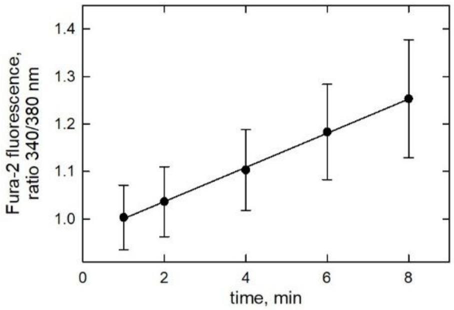

Figure S12. Cellular Fura-2 fluorescence intensity recorded from non-transfected HEK-cells immersed in MES assay buffer at pH 5.5 (MES 20 mM, NaCl 125 mM, $CaCl_2$ 0.8 mM). Shown are means (± s.d.) from three recordings at room temperature over time (slope of the regression line = 0.028 ratio units/min).

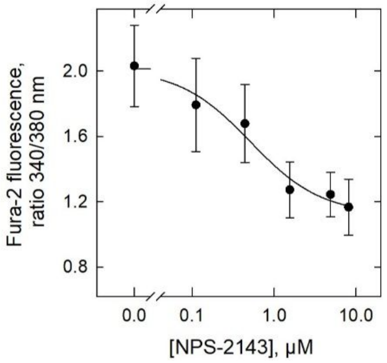

Figure S13. Inhibition of Ca^{2+}-mediated activation of the calcium-sensing receptor by NPS-2143. Four minutes after the addition of Ca^{2+} to a concentration of 3.8 mM, NPS-2143 was cumulatively added to establish the indicated concentrations. Four recordings with different concentrations NPS-2143 were carried out per cell-covered glass plate. DMSO, the solvent always was limited to a maximum of 0.3% the assay volume. Given are the means (± s.d.) of five recordings per data point.

Table S1

**Inactive**	**< 1.65**	**1.65 - 1.90**	**1.9 - 2.15**	**> 2.15**
1.11	1.34	1.69	1.95	2.27
1.20	1.55	1.82	1.91	2.16
1.09	1.28	1.75	2.12	2.24
1.17	1.16	1.76	2.10	2.33
	1.19	1.75	1.96	2.19
		1.75	1.97	2.30
		1.70	2.07	
			2.17	
			2.12	

Activation of the calcium-sensing receptor by individual thyroid drainage fluids after fractionation. Ratiometric values of Fura-2 fluorescence intensity recorded one minute after immersion of receptor-expressing cells into fraction four sample fluid. The values binned according to the Fura-2 fluorescence intensity range (first row of the Table) are depicted graphically in the histogram shown in Figure 4.
